# An open-source code to analyze mitochondrial intracellular distribution from fluorescence microscopy images

**DOI:** 10.1042/BSR20260140

**Published:** 2026-07-01

**Authors:** Giovanna C. Cavalcante, Alicia J. Kowaltowski

**Affiliations:** Departamento de Bioquímica, Instituto de Química, and Centro de Metabolismo CoMeta, Hospital Universitário, Universidade de São Paulo, São Paulo, Brazil

**Keywords:** Immunofluorescence, Mitochondria, Mitochondrial localization, Mitochondrial morphology, MitoTracker

## Abstract

Mitochondria have a plethora of roles in cells, many of which are related to dynamic changes in their size, shape, and intracellular location. Mitochondrial morphology is commonly assessed by microscopy with targeted fluorescent probes. However, tools to easily estimate mitochondrial localization within a cell are still lacking. A code was designed to estimate per-cell mitochondrial radial localization (perinuclear or peripheral) from fluorescence microscopy files in a variety of formats and using different mitochondrial markers (https://github.com/cavalcantegc/mito_localization.git). Three case studies with different cell types and stainings demonstrate that mitochondrial localization can be easily extracted and plotted with this code.

## Introduction

Mitochondria are cytoplasmic organelles crucial in energy metabolism and multiple other intracellular processes as diverse as controlling intracellular calcium, determining cell fate in differentiation, and maintaining redox balance. Thus, it is not surprising that mitochondrial dysfunctions are strongly linked to the development and progression of complex diseases, including metabolic disorders and neurodegenerative and cardiovascular diseases [1].

Among the myriad of mitochondrial characteristics that may influence function and behavior, mitochondrial morphology has attracted particular attention [2]. Morphology is a characteristic directly associated with mitochondrial dynamics, as abnormalities in fission and fusion processes can impact mitochondrial size and shape, as well as downstream positioning within the cell, influencing mitochondrial behavior [3]. For example, in brown adipose tissue, peri-lipid droplet mitochondria have more elongated morphology, low fission-fusion dynamics, low beta oxidation capacity and high ATP synthesis compared with cytoplasmic mitochondria [4]. Additionally, mitochondria located in the soma, dendrites, or axons of neurons display differences in function and morphology [5].

Recently, an association between mitochondrial localization in a cell and a variety of psychoses (bipolar disorder, schizophrenia, and schizoaffective disorder) has been uncovered [6]. In that study, a set of different fluorescence-based images (nuclei, cytoskeleton, and mitochondria) were processed using novel CellProfiler pipelines to obtain mitochondrial localization. Interestingly, mitochondrial localization seems to be distinct also in skin fibroblasts from patients with psychoses versus controls: mitochondria were found to be more internally localized, farther from the cell boundary, in the psychiatric patients. These findings reinforce the importance of mitochondrial cellular localization as a feature associated with functional outcomes.

## Hypothesis

To the best of our knowledge, there are no tools currently available to easily estimate mitochondrial localization in a cell using only typical fluorescence images, which we hypothesize would be of use for future research. We thus created an open-source Python code that estimates per-cell perinuclear and peripheral mitochondria from a variety of microscopy image types.

## Materials and methods

### Code design

**Figure 1:**
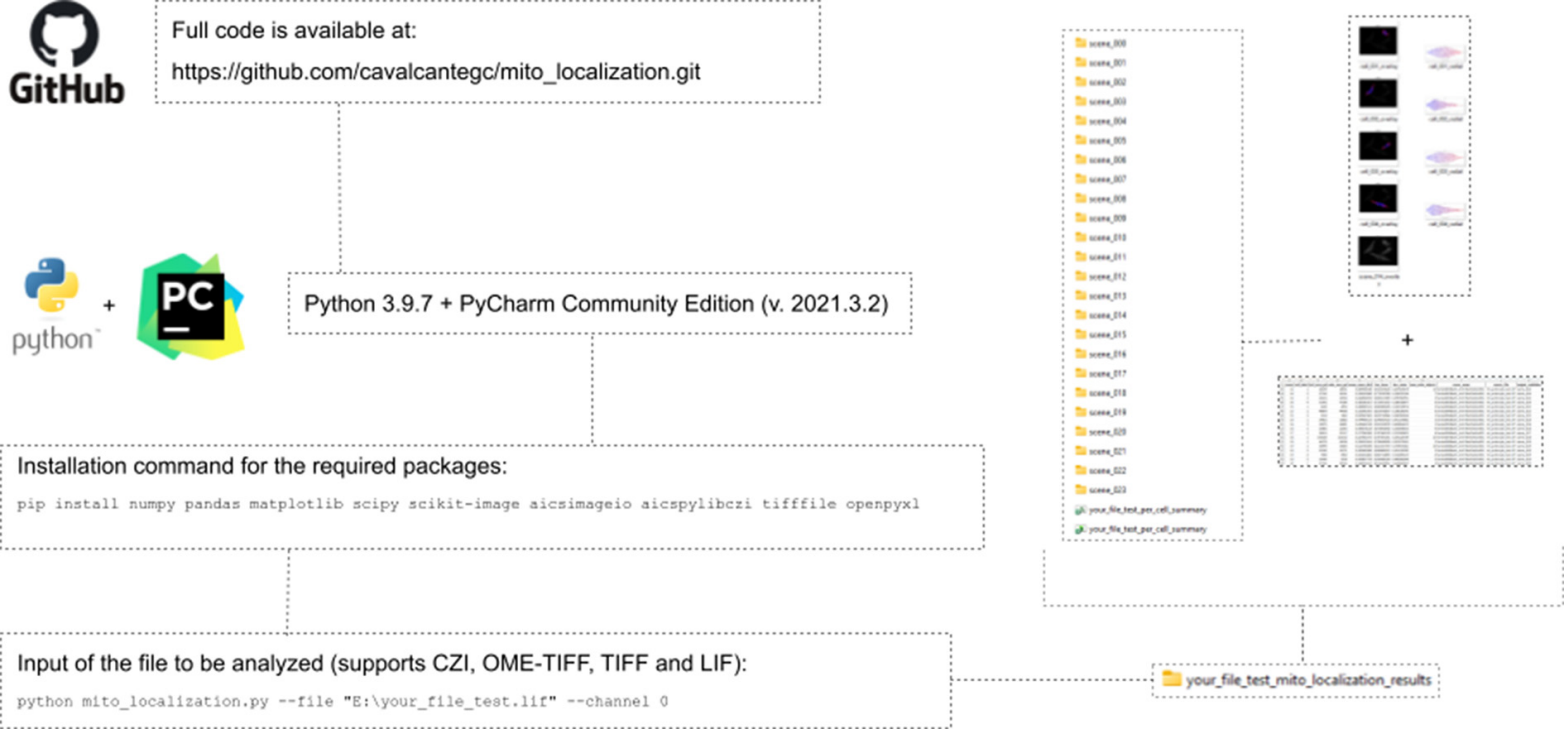
Workflow of the basic steps for code usage The simplified input system involves adding the location of the file to the PyCharm terminal, after which the user can run the designed code with a single command line after installing the required Python packages. More instructions are given throughout the code. User-friendly outputs include a folder with individual per-cell data of each image and a summary spreadsheet.

A code was designed to estimate per-cell mitochondrial radial localization (perinuclear or peripheral) from fluorescence microscopy assays with mitochondrial staining, using Python 3.9.7 programming language in the PyCharm Community Edition (v. 2021.3.2) environment. The full code is openly available at https://github.com/cavalcantegc/mito_localization.git, and Figure 1 shows a step-by-step workflow for code usage.

The script estimates cell outlines from the fluorescence signal (stainings such as MitoTracker probes) and reads one or more images (micrographs captured in the assay) in each input file in a single run. It supports CZI, OME-TIFF, TIFF, and LIF formats as input and any staining that marks individualized mitochondria. The code was designed to generate a directory with folders for each image, including PNG violin plots (one per cell) showing the radial distribution of the mitochondrial area and an overlay for each cell. It also generates a spreadsheet with the summary of per-cell metrics, including the fraction of inner (perinuclear) and outer (peripheral) mitochondria in each labeled cell, which can then be exported to other data analysis software. All outputs are saved in an output folder next to the input file in the directory.

#### Algorithmic steps

Each scene was read using AICSImageIO and converted to a 2D maximum-intensity projection (if Z-stacks were present), followed by minimum–maximum normalization to 8-bit. Cell boundaries are estimated directly from the mitochondrial signal. Images are Gaussian-smoothed (default σ = 2.0) and thresholded using Otsu’s method (fallback: mean + 1 SD). Small objects are removed (minimum cell area = 2000 pixels), followed by binary closing (5 × 5 structuring element) and hole filling to approximate full cytoplasmic area. Connected components are labeled as individual cells. Touching cells are not split by watershed in the default implementation; merged regions are treated as single cells.

### Applications

To demonstrate the functionality of our Python code to quantify mitochondrial intracellular distribution, we first tested data acquired by our group using human astrocytes. The code was applied to analyze a *lif* file with multiple images from assays of the cells stained with MitoTracker Deep Red FM (Invitrogen, #M22426), which accumulates in the mitochondria of living cells in an inner membrane potential-dependent manner. The nucleus area was estimated from the ‘void’ in the captured cell. Photographs were obtained using a Leica DMi8 inverted microscope with 63x zoom.

To expand the code usage to *czi* files, we also applied it to immunofluorescence micrograph files of mouse embryonic fibroblasts (MEFs) kindly donated by the Scorrano group at the University of Padova, Italy. Mitochondria are marked as indicated by mitochondrial protein SDHA (succinate dehydrogenase complex flavoprotein subunit A). Photographs were obtained with the Zeiss LSM900 Airyscan confocal microscope with 63× zoom.

Moreover, we also accessed publicly available datasets with microscopy images from the CRBS Cell Image Library (RRID:SCR_003510). We applied the code to the *tif* file under accession number 13731, from a study by Jin et al. [7]. The image shows HeLa cells expressing mito-YFP (green), localized to mitochondria, and stained for Tom20 (white), a mitochondrial outer membrane protein, resulting in two different fluorophores marking mitochondria. Photographs were obtained with the Zeiss LSM510 Meta microscope with a 63× zoom.

## Results and discussion

A common assay to visualize mitochondrial morphology is staining with fluorescent probes such as MitoTracker, generating images that can be analyzed by open software such as ImageJ, which has add-ons capable of generating automated quantifications of mitochondrial characteristics such as sphericity and mean volume per cell. However, subcellular mitochondrial location could not be easily analyzed with tools already at hand.

**Figure 2:**
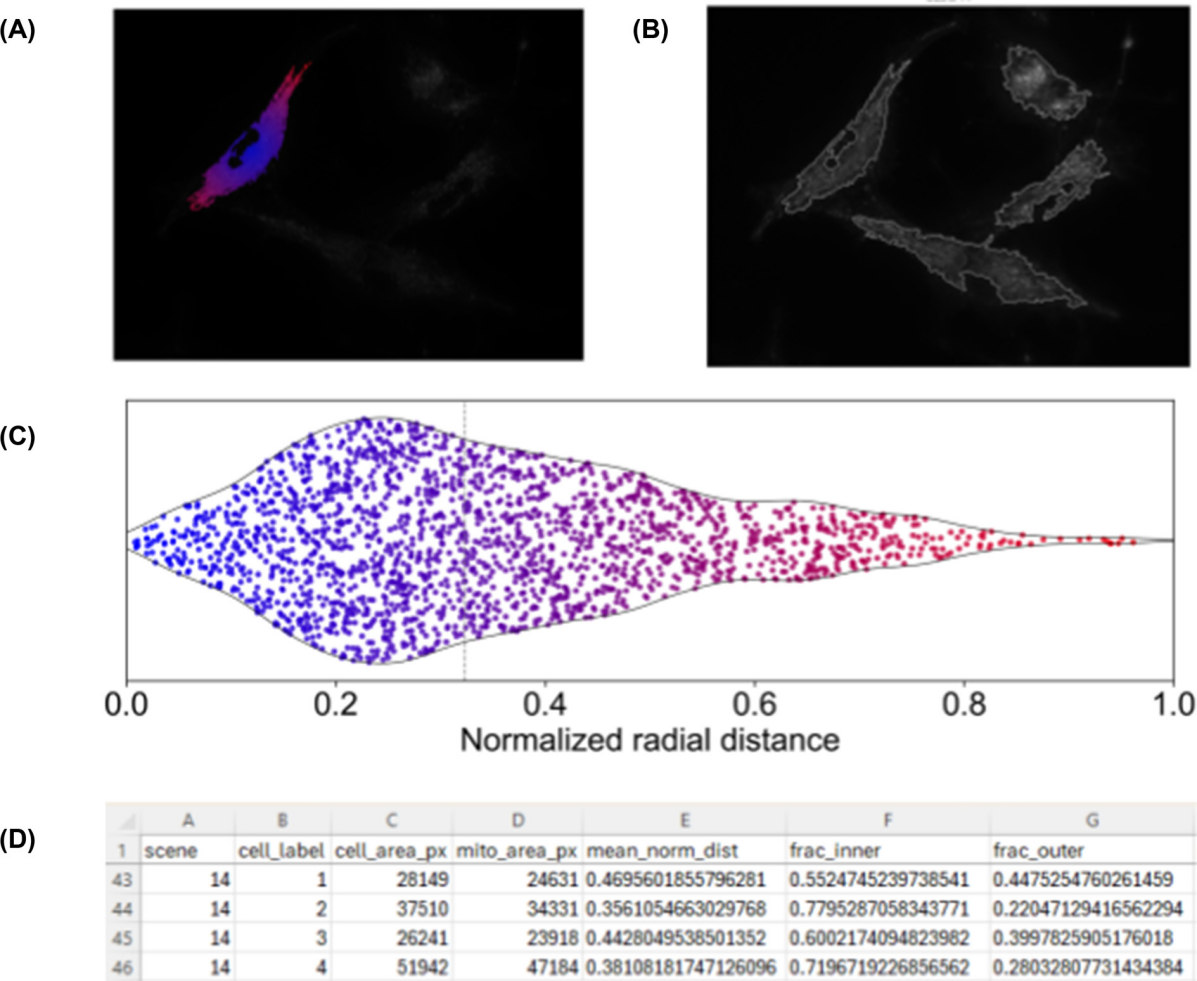
Mitochondrial localization quantification in human astrocytes (**A**) Per-cell estimates of mitochondrial localization show a color gradient from perinuclear (blue) to peripheral (red) mitochondria obtained from analysis of a MitoTracker-stained cell image (gray). (**B**) Gray layer of cells detected and mitochondria in the image. In this example, ‘Scene 14’ shows four cells. (**C**) Violin plot with the fraction of mitochondrial pixels (dots) identified from perinuclear (center, 0, in blue) to peripheral (periphery, 1, in red). (**D**) Values in the spreadsheet generated for the four cells in Panel B.

Considering this gap, we developed a user-friendly Python code to estimate per-cell mitochondrial radial localization (perinuclear or peripheral) from *lif* image files with mitochondria-targeted fluorescence, such as MitoTracker. The code produces a spreadsheet with per-cell measurements and visual outputs, which facilitate the visualization of mitochondrial distribution both by using a color code (Figure 2A) and by quantifying distance from the nucleus (Figure 2C). With a single command line as input, the code identified each cell (Figure 2B) and estimated the fraction of mitochondria that are in inner and outer portions of the cell, depicting the data as figures and quantitative spreadsheets (Figure 2D). In the representative cell depicted in Figure 2, more mitochondria are perinuclear, although a wide variety of locations is observed. Mitochondrial dynamics, particularly fission, may modulate morphogenesis and mitochondrial organization within the cell in astrocytes [8], highlighting the importance of being able to readily quantify mitochondrial distribution in a cell. The scene in Figure 2 comes from a *lif* file with 12 scenes, each one containing at least one astrocyte. In this first example, the code was applied to nearly 400 scenes.

**Figure 3:**
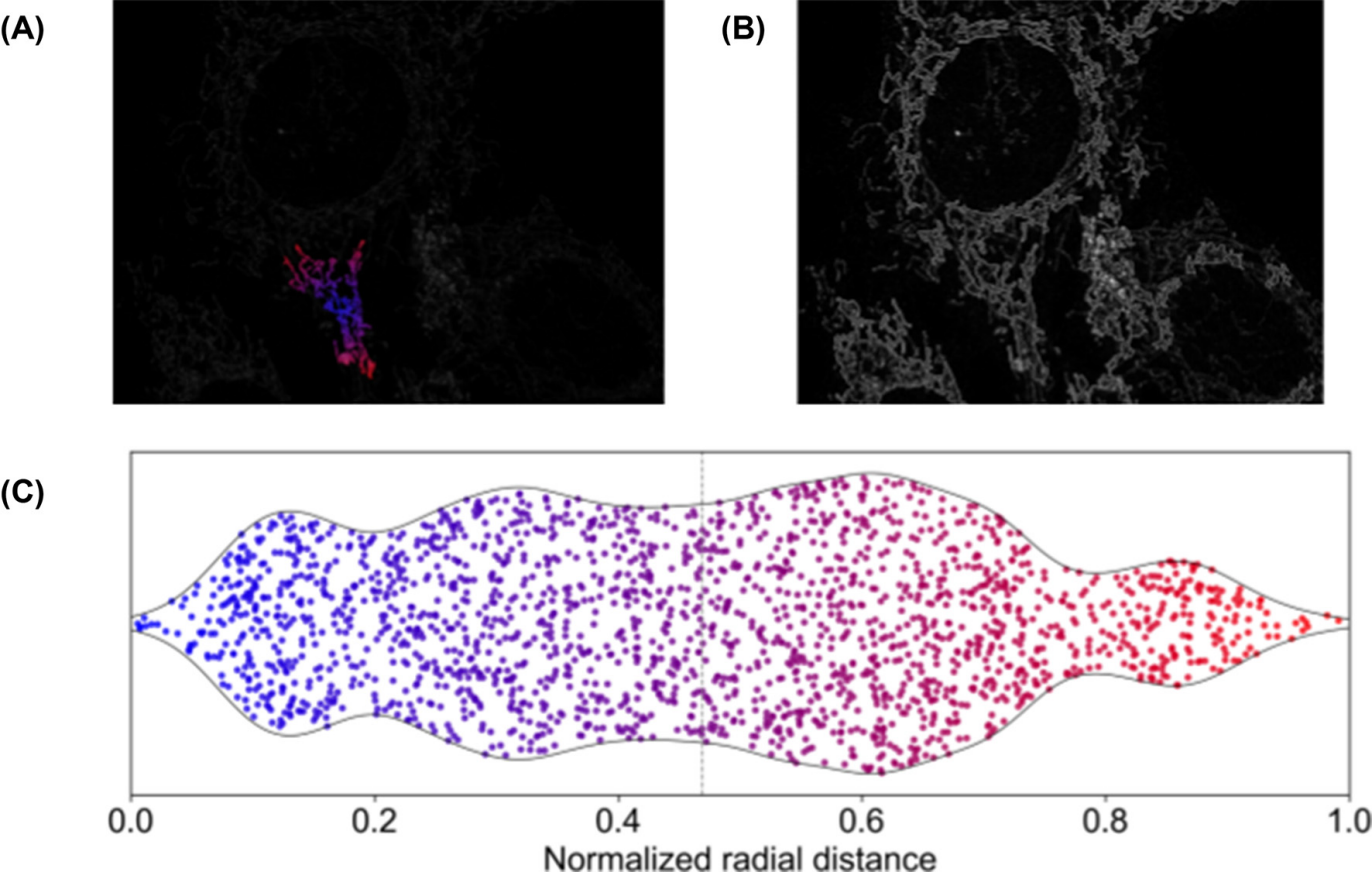
Mitochondrial localization in MEFs (**A**) Estimates of mitochondrial localization in a cell-cell contact network from center (blue) to periphery (red) from the anti-SDHA immunofluorescence (gray). (**B**) Gray layer of cells and mitochondria detected in the image file. (**C**) Violin plot with the fraction of mitochondrial pixels (dots) identified from center (0, in blue) to periphery (1, in red) for the highlighted region.

In the second example, we applied the code to three *czi* files in which MEF cells were stained with anti-SDHA immunofluorescence to test the code for use with different mitochondrial stains and image file types. No issues were observed when changing either the file format or the fluorescence modality, and the code was executed successfully. However, the test showed that when using a different cell type, adjustments were needed. MEFs are more rounded than astrocytes, with larger nucleus ‘voids’ and sparse mitochondria forming clusters. In this image, MEFs are also touching each other, forming closer mitochondrial networks, as seen in Figure 3. Sparse mitochondrial signals can cause oversegmentation, while physically adjacent cells may be perceived as a single cell. This highlights that, as with other automated detection codes, visual checks for quality control of the cell separations and mitochondrial identification are important. Additionally, given the nature of the code is to determine the location of individual mitochondria within cells, it requires cells with at least a partially fragmented mitochondrial network.

**Figure 4:**
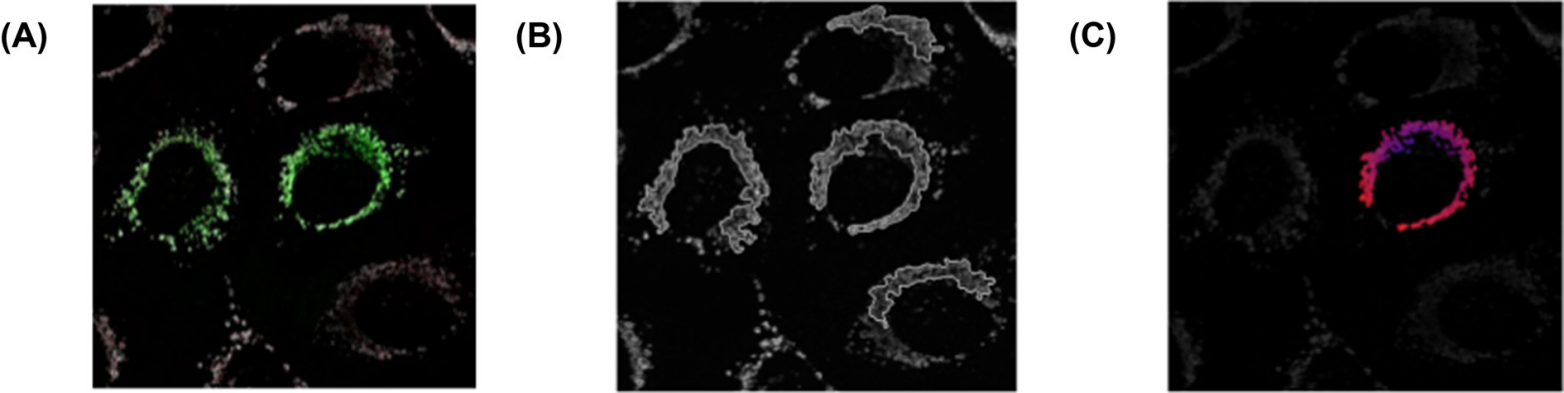
Mitochondrial localization in HeLa cells (**A**) Original *tif* figure showing HeLa cells colored by mito-YFP (green) and Tom20 (white) in the same channel. (**B**) Gray layer of detected cells and mitochondria for both fluorophores in the single image of the file. The brightest parts of the cells are correctly detected. (**C**) Per-cell estimates of mitochondrial localization from center (blue) to periphery (red).

A third case study of the code consisted of the application to a *tif* file of HeLa cells expressing mito-YFP and stained for TOM20 from the public repository CLI under accession number 13731. We also applied the code to 10 other *tif* files, with different types of cells and staining, from the same public repository. We found that the code can automatically recognize mitochondria even when multiple mitochondria-targeted probes are mixed within the same channel (Figure 4).

Overall, the code performed well as a tool to assess mitochondrial distribution. Our tests indicated that the code infers cell boundaries exclusively from mitochondrial fluorescence, requiring only dense and broadly distributed mitochondrial labeling to approximate cellular area, as is typical of most cell types. It performs reliably with mitochondrial matrix–localized reporters, MitoTracker probes, or outer mitochondrial membrane markers that provide homogeneous cytoplasmic coverage. It does not require nuclear or membrane markers, nor a necessary change of code for every individual analysis. The code primarily works for images of spread, adherent cells with distributed mitochondrial networks, while touching cells may be segmented as a single cell in the absence of an independent nuclear or cytoplasmic reference channel. In such cases, images should be excluded. Consequently, the code is best suited for mitochondria-centric analyses rather than general-purpose cell segmentation.

## Future directions

We hope that this automated and quantitative identification of mitochondrial cellular location is useful in a number of studies that involve generating images with mitochondrial markers and can contribute toward better understanding of mitochondrial biology in health and disease.

## Conclusions

We successfully generated a code capable of identifying and separating by peripheral or central location mitochondria within cells in images of different formats and with different mitochondrial stains.

## Data Availability

The open-source code developed here was made fully available at https://github.com/cavalcantegc/mito_localization.git [9]. Raw files are made available upon reasonable request.

## Abbreviations

MEFs: mouse embryonic fibroblasts
SDHA: succinate dehydrogenase complex flavoprotein subunit A
TOM20: translocase of outer mitochondrial membrane 20
